# Supplementary material for: Neocortical localization and thalamocortical modulation of neuronal hyperexcitability contribute to Fragile X Syndrome
Source: Commun Biol. 2022 May 11;5:442. doi: 10.1038/s42003-022-03395-9 (PMC9095835; doi:10.1038/s42003-022-03395-9)
Supplement: Supplementary file 2 — Supplementary Information [file 42003_2022_3395_MOESM2_ESM.pdf]

**Neocortical Localization and Thalamocortical Modulation of Neuronal Hyperexcitability contribute to Fragile X Syndrome**

**Supplementary Figure 1. Group Contrasts of Topographical Power of Scalp EEG by Frequency Band**

Group-level t-maps (5% FDR-corrected) of cluster permutation statistical comparison between FXS (n=70) and TDC (n=71) of scalp (electrode-level) EEG power As findings across scalp EEG remain spatially ambiguous[1], the findings were primarily used to generate hypotheses and assess for frequency specific trends across groups. Warmer colors indicate significant FXS>TDC, cooler colors indicate significant FXS<TDC, and gray areas indicate non-significant group differences.

a. Relative Power (FXS-TDC)

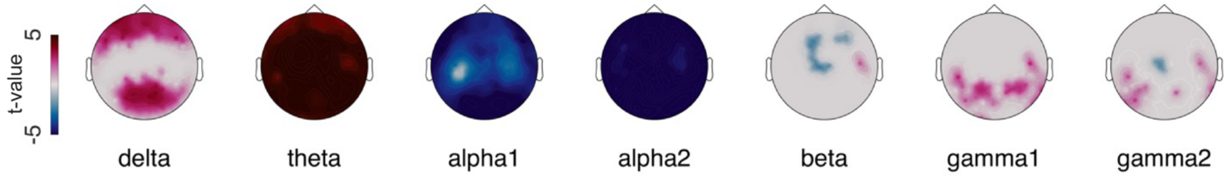


b. Absolute Power (FXS-TDC)

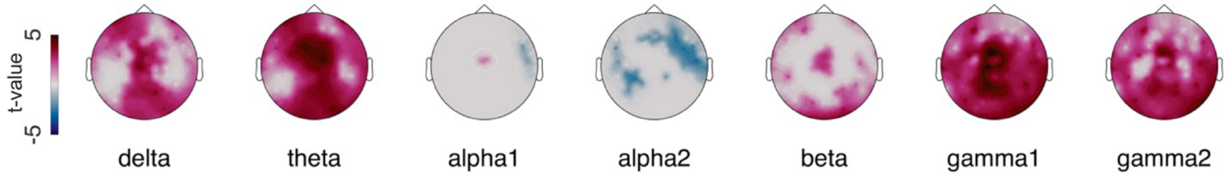

**Supplementary Figure 2. Group Contrasts of Source-level Absolute Power by Frequency Band**

Group level t-maps depicting FXS – Control, vertex-by-vertex absolute power differences by frequency band superimposed on brain surface models. Warmer (FXS > Control) and cooler (FXS < Control) color scale represents significant t-values (non-significant values as gray).

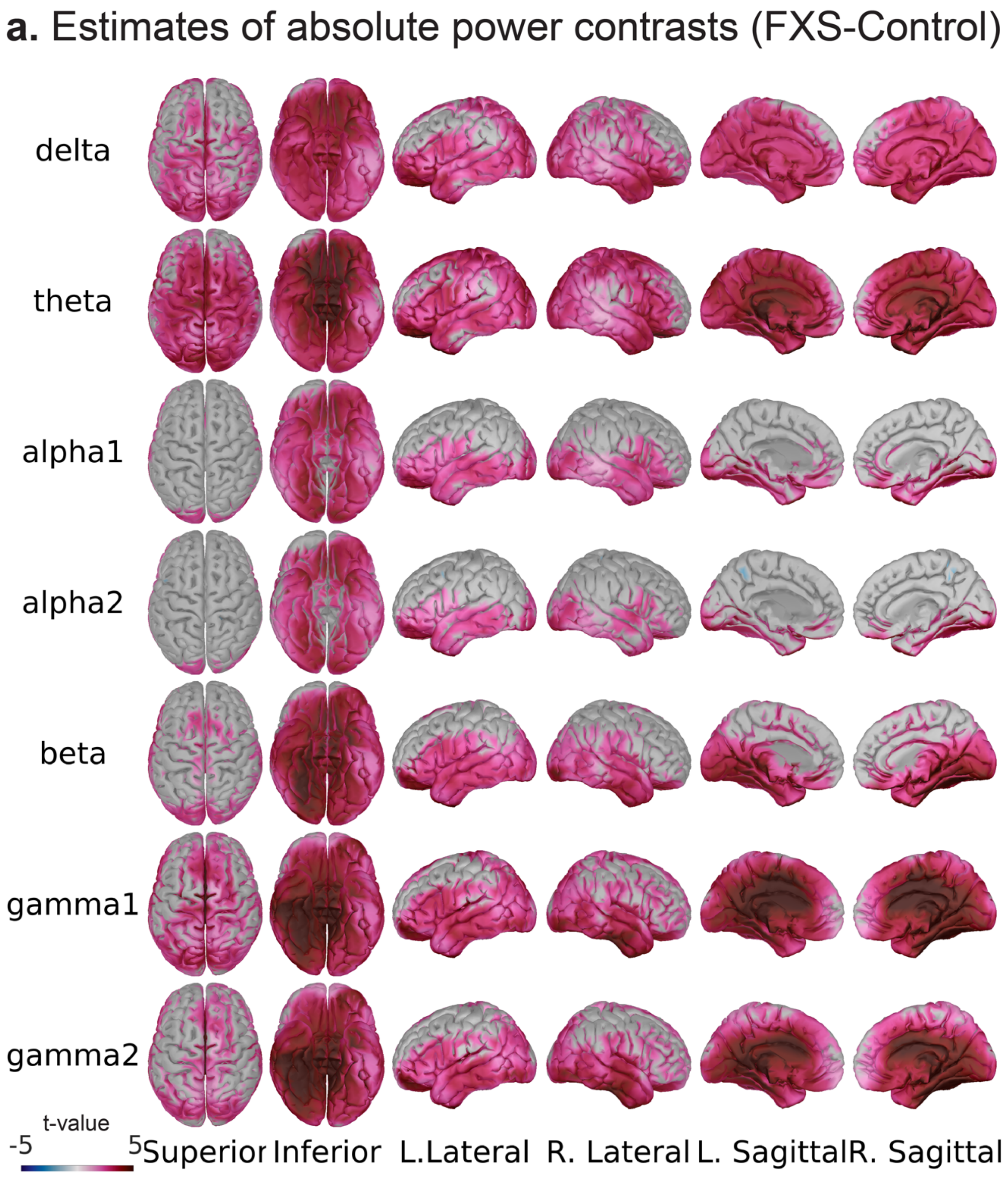

**Supplementary Figure 3. Node-level parcellation key for Desikan-Killiany Cortical Atlas.**

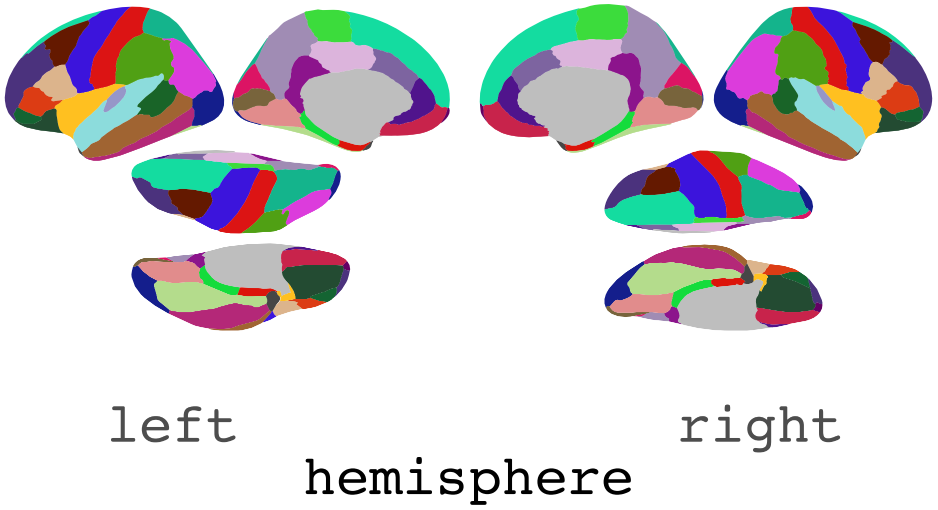


Desikan-Killany Atlas Nodes

- bankssts
- caudal anterior cingulate
- caudal middle frontal
- corpus callosum
- cuneus
- entorhinal
- fusiform
- inferior parietal
- inferior temporal
- isthmus cingulate
- lateral occipital
- lateral orbitofrontal
- lingual
- medial orbitofrontal
- middle temporal
- parahippocampal
- paracentral
- pars opercularis
- pars orbitalis
- pars triangularis
- pericalcarine
- postcentral
- posterior cingulate
- precentral
- precuneus
- rostral anterior cingulate
- rostral middle frontal
- superior frontal
- superior parietal
- superior temporal
- supramarginal
- frontal pole
- temporal pole
- transverse temporal
- insula

**Supplementary Table 1. Additional Clinical Characteristics by Group**

Comparison of detailed demographic and clinical features of EEG dataset by group. FSIQ, Full Scale IQ, NVIQ, Non-verbal intelligence scale; VIQ, verbal intelligence scale; SCQ, Social Communication Questionnaire; WJ-3, Woodcock III Tests of Cognitive Abilities; ABC, Aberrant Behavioral Checklist; ADAMS, Anxiety, Depression, and Mood Scale; t, t-statistic following independent Student t-tests; p, unadjusted significance; adj. p, p-value following Bonferroni correction.

| | **Control(M)** | **FXS(M)** | **Control(F)** | **FXS(F)** | **p.overall** |
|---|---|---|---|---|---|
| | ***N=41*** | ***N=38*** | ***N=30*** | ***N=32*** | |
| Age (Years) | 22.2 (9.81) | 22.7 (9.95) | 22.0 (12.0) | 17.9 (9.61) | 0.200 |
| FSIQ | 106 (10.1) | 34.5 (22.8) | 99.9 (6.78) | 65.5 (29.2) | <0.001 |
| VIQ | 108 (13.3) | 46.9 (24.2) | 97.0 (9.38) | 71.7 (27.9) | <0.001 |
| NVIQ | 104 (12.2) | 22.1 (28.4) | 103 (8.65) | 59.2 (32.9) | <0.001 |
| SCQ | 1.73 (1.93) | 17.1 (6.49) | 2.65 (2.52) | 9.36 (7.00) | <0.001 |
| ABC-Irritability | 0.47 (0.97) | 11.5 (9.30) | 1.12 (2.66) | 7.21 (7.87) | <0.001 |
| ABC-Hyperactivity | 0.90 (1.21) | 18.3 (11.3) | 1.25 (4.23) | 8.21 (8.02) | <0.001 |
| ABC-Abnormal Speech | 0.10 (0.31) | 5.88 (3.29) | 0.19 (0.75) | 2.50 (2.74) | <0.001 |
| ABC-Lethargy | 1.30 (3.23) | 8.39 (6.76) | 0.81 (2.48) | 7.50 (8.92) | <0.001 |
| ABC-Stereotypy | 0.03 (0.18) | 6.30 (5.56) | 0.12 (0.50) | 1.86 (3.82) | <0.001 |
| ADAMS-OCD | 0.23 (0.68) | 3.14 (2.66) | 0.56 (1.54) | 1.29 (1.84) | <0.001 |
| ADAMS-Anxiety | 2.03 (2.22) | 7.83 (5.01) | 2.28 (3.12) | 6.37 (5.07) | <0.001 |
| WJ-III | 95.3 (13.4) | 65.5 (15.8) | 91.3 (8.02) | 70.9 (15.4) | <0.001 |

**Supplementary Table 2. Preprocessing.** Statistical comparison of preprocessing characteristics demonstrating no significant group differences in key artifact cleaning routines across final datasets.

| | **FXS** *N=70* | **Control** *N=71* | **p** |
|---|---|---|---|
| Remaining Trials | 125 (24.6) | 131 (18.1) | 0.117 |
| Total Duration(s) | 315 (29.0) | 311 (25.9) | 0.437 |
| Clean Duration(s) | 250 (49.2) | 262 (36.1) | 0.115 |
| Bad Channels | 6.27 (3.31) | 5.93 (3.71) | 0.564 |
| Artifact Components | 11.3 (1.71) | 10.8 (2.58) | 0.117 |

**Supplementary Table 3. Summary of Source-estimated Peak Alpha Frequency (PAF) by Cortical Node.** A linear mixed effect model was conducted to examine the effect of group, sex, and cortical node location on source estimated PAF (subject was a random effect). A significant interaction effect between group and node (group x node; $F_{67,9378}=1.44$, $p=0.01$) was present, but no effect of sex. Each row summarizes 5% FDR corrected significant pairwise comparisons (FXS-Control) of PAF estimates (Hz). Atlas key in Appendix 1. Abbreviations: FXS, Fragile X Syndrome; Cont., control; SE, standard error; DF, degrees of freedom; FDR, false discovery rate.

| Node | Region | FXS | TDC | FXS-TDC | SE | DF | F | 5% FDR |
|---|---|---|---|---|---|---|---|---|
| Banks of Sup. Temp. Sulcus R | RT | 8.24±.22 | 9.19±.22 | -.95 | .30 | 137 | -3.1 | 0.011 |
| Caudal Anterior Cingulate L | LL | 7.96±.22 | 8.72±.22 | -.76 | .31 | 137 | -2.5 | 0.037 |
| Caudal Anterior Cingulate R | RL | 7.74±.22 | 8.61±.22 | -.87 | .30 | 137 | -2.8 | 0.017 |
| Caudal Middle Frontal L | LF | 8.26±.22 | 9.29±.22 | -1.03 | .31 | 137 | -3.4 | 0.008 |
| Caudal Middle Frontal R | RF | 8.39±.22 | 9.21±.22 | -.82 | .30 | 137 | -2.7 | 0.024 |
| Cuneus L | LO | 7.86±.22 | 8.79±.22 | -.93 | .30 | 137 | -3.1 | 0.011 |
| Cuneus R | RO | 8.45±.22 | 9.46±.22 | -1.01 | .30 | 137 | -3.3 | 0.009 |
| Entorhinal L | LT | 7.61±.22 | 8.34±.22 | -.73 | .30 | 137 | -2.4 | 0.045 |
| Fusiform R | RT | 8.54±.22 | 9.28±.22 | -.74 | .30 | 137 | -2.4 | 0.041 |
| Inferior Parietal L | LP | 8.30±.22 | 9.26±.22 | -.97 | .30 | 137 | -3.2 | 0.011 |
| Insula L | LT | 8.29±.22 | 9.19±.22 | -.91 | .30 | 137 | -3.0 | 0.012 |
| Isthmus Cingulate L | LL | 8.40±.22 | 9.33±.22 | -.93 | .30 | 137 | -3.0 | 0.011 |
| Isthmus Cingulate R | RL | 8.27±.22 | 9.26±.22 | -.99 | .30 | 137 | -3.3 | 0.010 |
| Lateral Occipital L | LO | 8.22±.22 | 9.13±.22 | -.92 | .30 | 137 | -3.0 | 0.011 |
| Paracentral L | LC | 8.88±.22 | 9.60±.22 | -.72 | .30 | 137 | -2.4 | 0.045 |
| Paracentral R | RC | 8.68±.22 | 9.46±.22 | -.78 | .30 | 137 | -2.5 | 0.034 |
| Pars Opercularis L | LF | 7.79±.22 | 8.51±.22 | -.72 | .30 | 137 | -2.4 | 0.045 |
| Pars Triangularis R | RF | 7.68±.22 | 8.79±.22 | -1.12 | .30 | 137 | -3.7 | 0.004 |
| Postcentral L | LC | 8.25±.22 | 9.48±.22 | -1.23 | .30 | 137 | -4.0 | 0.002 |
| Postcentral R | RC | 8.31±.22 | 9.23±.22 | -.92 | .30 | 137 | -3.0 | 0.011 |
| Posteriorcingulate L | LL | 8.23±.22 | 9.17±.22 | -.94 | .31 | 137 | -3.1 | 0.011 |
| Posteriorcingulate R | RL | 8.00±.22 | 8.95±.22 | -.95 | .31 | 137 | -3.1 | 0.011 |
| Precentral L | LC | 8.19±.22 | 9.62±.22 | -1.43 | .30 | 137 | -4.7 | <0.001 |
| Precentral R | RC | 8.51±.22 | 9.26±.22 | -.75 | .31 | 137 | -2.4 | 0.041 |
| Precuneus L | LP | 8.07±.22 | 9.34±.22 | -1.27 | .30 | 137 | -4.2 | 0.001 |
| Precuneus R | RP | 8.31±.22 | 9.10±.22 | -.79 | .30 | 137 | -2.6 | 0.033 |
| Superior Parietal L | LP | 8.11±.22 | 9.26±.22 | -1.15 | .30 | 137 | -3.8 | 0.003 |
| Superior Parietal R | RP | 7.85±.22 | 9.21±.22 | -1.36 | .30 | 137 | -4.5 | <0.001 |
| Supramarginal L | LP | 8.72±.22 | 9.82±.22 | -1.10 | .30 | 137 | -3.6 | 0.004 |
| Supramarginal R | RP | 8.34±.22 | 9.30±.22 | -.95 | .30 | 137 | -3.1 | 0.011 |

**Supplementary Table 4. Pairwise group differences of gamma1 cross-frequency coupling by cortical node.**

| Pairwise (FXS-Control) gamma1 power-power CFC comparisons by cortical node | | | | | | |
|---|---|---|---|---|---|---|
| | Theta | | Alpha1 | | Alpha2 | |
| Node | L | R | L | R | L | R |
| Banks of Sup. Temp. Sulcus | -.08* | -.12*** | .06* | .06* | .10** | .06* |
| Caudal Anterior Cingulate | -.11** | -.11** | .02 | .02 | .08* | .08* |
| Caudal Middle Frontal | -.11** | -.11*** | .03 | .03 | .07* | .05 |
| Cuneus | -.05* | -.09* | .06* | .08* | .09* | .12*** |
| Entorhinal | -.08* | -.05 | .06* | .08* | .08* | .10** |
| Frontal Pole | .02 | .01 | .05* | .06* | .06* | .06* |
| Fusiform | -.06* | -.07* | .08* | .09* | .09* | .12*** |
| Inferior Parietal | -.08* | -.11** | .04 | .07* | .10** | .08* |
| Inferior Temporal | -.08* | -.07* | .04 | .09* | .07* | .10** |
| Insula | -.08* | -.05* | .04 | .03 | .05 | .07* |
| Isthmus Cingulate | -.12*** | -.13*** | .10** | .11** | .15*** | .12*** |
| Lateral Occipital | -.03 | -.05* | .08* | .07* | .09* | .09* |
| Lateral Orbitofrontal | -.01 | -.04 | .11** | .06* | .10** | .09** |
| Lingual | -.03 | -.04 | .12*** | .09* | .08* | .09** |
| Medial Orbitofrontal | -.01 | -.05 | .08* | .06* | .08* | .08* |
| Middle Temporal | -.06* | -.06* | .07* | .07* | .07* | .10** |
| Paracentral | -.12*** | -.12*** | .04 | .03 | .12*** | .11*** |
| Parahippocampal | -.08* | -.09* | .09** | .09** | .09** | .11** |
| Pars Opercularis | -.06* | -.03 | .02 | .05 | .06* | .05* |
| Pars Orbitalis | -.02 | .03 | .07* | .03 | .08* | .05 |
| Pars Triangularis | -.05* | -.06* | .04 | .01 | .07* | .06* |
| Pericalcarine | -.03 | -.04 | .11** | .09** | .08* | .07* |
| Postcentral | -.12*** | -.10** | .06* | .05 | .07* | .05* |
| Posteriorcingulate | -.13*** | -.13*** | .04 | .05 | .10** | .09* |
| Precentral | -.11*** | -.13*** | .06* | .05 | .06* | .08* |
| Precuneus | -.12*** | -.14*** | .09* | .06* | .10** | .11*** |
| Rostral Anterior Cingulate | -.03 | -.03 | .07* | .06* | .06* | .07* |
| Rostral Middle Frontal | -.03 | -.04 | .06* | .02 | .06* | .05* |
| Superior Frontal | -.09** | -.08* | .01 | -.02 | .07* | .07* |
| Superior Parietal | -.13*** | -.13*** | .09* | .09* | .07* | .11** |
| Superior Temporal | -.07* | -.06* | .05 | .06* | .06* | .09* |
| Supramarginal | -.11** | -.10** | .05* | .04 | .08* | .06* |
| Temporal Pole | -.04 | -.05 | .05 | .08* | .05* | .10** |
| Transverse Temporal | -.08* | -.08* | .07* | .05* | .07* | .09** |

**Supplementary Table 5. Pairwise group differences of gamma1 cross-frequency coupling by resting state network (RSN).**

| RSN | Lower Band | Estimate | Statistic | DF | 5% FDR p | Sig. |
|---|---|---|---|---|---|---|
| DMN | theta | -.08±.01 | -6.26 | 139 | 1.6e-08 | *** |
| | alpha1 | .07±.01 | 5.62 | 139 | 2.3e-07 | *** |
| | alpha2 | .09±.01 | 7.34 | 139 | 2.9e-10 | *** |
| DAN | theta | -.05±.01 | -3.91 | 139 | 2.0e-04 | *** |
| | alpha1 | .05±.01 | 3.48 | 139 | 8.1e-04 | *** |
| | alpha2 | .07±.01 | 5.41 | 139 | 5.4e-07 | *** |
| SAN | theta | -.08±.01 | -5.64 | 139 | 2.3e-07 | *** |
| | alpha1 | .04±.01 | 2.65 | 139 | 9.0e-03 | ** |
| | alpha2 | .06±.01 | 4.37 | 139 | 3.9e-05 | *** |
| AUD | theta | -.07±.02 | -3.41 | 139 | 9.6e-04 | *** |
| | alpha1 | .05±.02 | 2.82 | 139 | 5.8e-03 | ** |
| | alpha2 | .08±.02 | 3.96 | 139 | 1.8e-04 | *** |
| VIS | theta | -.05±.01 | -3.79 | 139 | 2.9e-04 | *** |
| | alpha1 | .08±.01 | 5.86 | 139 | 9.6e-08 | *** |
| | alpha2 | .09±.01 | 6.80 | 139 | 2.6e-09 | *** |
| other | theta | -.08±.01 | -6.49 | 139 | 8.5e-09 | *** |
| | alpha1 | .06±.01 | 4.65 | 139 | 1.4e-05 | *** |
| | alpha2 | .08±.01 | 6.38 | 139 | 1.1e-08 | *** |

**Supplementary Table 6. Age-corrected Clinical Correlations of Spectral Power by Cortical Region**

Abbreviations: See Appendix 2 for region atlas key; ABC, Aberrant Behavior Checklist; ADAMS, Anxiety, Depression and Mood Scale; SCQ, Social Communication Questionnaire; VIQ, verbal intelligence quotient.

**All FXS Participants**

| | | Spearman's ρ | | | | Age-Corrected | |
|---|---|---|---|---|---|---|---|
| Measure | Frequency | Region | n | rho | adj.p | rho | adj.p |
| ABC-Abnormal Speech | alpha1 | LPF | 61 | -.44 | 0.01 | -.48 | <0.01 |
| | | RPF | 61 | -.40 | 0.03 | -.44 | 0.02 |
| | alpha2 | LPF | 61 | -.35 | 0.05 | -.38 | 0.04 |
| ABC-Hyperactivity | alpha1 | | 61 | -.42 | 0.02 | -.40 | 0.03 |
| | | LT | 61 | -.46 | 0.01 | -.44 | 0.02 |
| | | RPF | 61 | -.44 | 0.01 | -.42 | 0.02 |
| | | RT | 61 | -.49 | <0.01 | -.48 | <0.01 |
| ABC-Stereotypy | theta | LPF | 61 | -.40 | 0.03 | -.40 | 0.03 |
| | | RPF | 61 | -.39 | 0.03 | -.39 | 0.04 |
| | alpha1 | LO | 61 | -.30 | 0.1 | -.39 | 0.04 |
| | | LPF | 61 | -.47 | <0.01 | -.49 | <0.01 |
| | | LT | 61 | -.32 | 0.08 | -.39 | 0.04 |
| | | RO | 61 | -.33 | 0.07 | -.41 | 0.03 |
| | | RPF | 61 | -.48 | <0.01 | -.50 | <0.01 |
| | | RT | 61 | -.38 | 0.04 | -.45 | 0.01 |
| | alpha2 | LPF | 61 | -.44 | 0.01 | -.46 | 0.01 |
| | | RO | 61 | -.32 | 0.08 | -.39 | 0.04 |
| | | RPF | 61 | -.39 | 0.03 | -.41 | 0.03 |
| ADAMS-OCD | alpha1 | LO | 64 | -.39 | 0.03 | -.42 | 0.02 |
| | | LP | 64 | -.36 | 0.04 | -.39 | 0.03 |
| | | LPF | 64 | -.39 | 0.03 | -.39 | 0.03 |
| | | LT | 64 | -.44 | 0.01 | -.47 | <0.01 |
| | | RO | 64 | -.36 | 0.05 | -.38 | 0.04 |
| | | RP | 64 | -.36 | 0.04 | -.39 | 0.03 |
| | | RPF | 64 | -.43 | 0.01 | -.44 | 0.02 |
| | | RT | 64 | -.45 | <0.01 | -.48 | <0.01 |
| | alpha2 | LC | 64 | -.39 | 0.03 | -.39 | 0.03 |
| | | LL | 64 | -.49 | <0.01 | -.49 | <0.01 |
| | | LP | 64 | -.40 | 0.03 | -.42 | 0.02 |
| | | LPF | 64 | -.45 | <0.01 | -.45 | 0.01 |
| | | LT | 64 | -.40 | 0.02 | -.42 | 0.02 |
| | | RC | 64 | -.40 | 0.02 | -.40 | 0.03 |
| | | RL | 64 | -.48 | <0.01 | -.48 | <0.01 |
| | | RP | 64 | -.42 | 0.02 | -.43 | 0.02 |
| | | RPF | 64 | -.44 | 0.01 | -.44 | 0.01 |
| | | RT | 64 | -.42 | 0.02 | -.44 | 0.02 |
| SCQ | alpha1 | LPF | 62 | -.46 | <0.01 | -.48 | <0.01 |
| | | LT | 62 | -.35 | 0.06 | -.41 | 0.03 |
| | | RPF | 62 | -.46 | <0.01 | -.49 | <0.01 |
| | | RT | 62 | -.33 | 0.07 | -.39 | 0.04 |
| | alpha2 | LF | 62 | -.38 | 0.03 | -.39 | 0.04 |
| | | LL | 62 | -.40 | 0.03 | -.42 | 0.02 |
| | | LPF | 62 | -.51 | <0.01 | -.52 | <0.01 |
| | | RC | 62 | -.36 | 0.05 | -.37 | 0.05 |
| | | RL | 62 | -.40 | 0.03 | -.43 | 0.02 |
| | | RPF | 62 | -.47 | <0.01 | -.49 | <0.01 |
| VIQ | alpha1 | LPF | 64 | .35 | 0.05 | .40 | 0.03 |

**Supplementary Table 7. Age-corrected Clinical Correlations of Spectral Power by Cortical Region**

Abbreviations: See Appendix 2 for region atlas key; ABC, Aberrant Behavior Checklist; ADAMS, Anxiety, Depression and Mood Scale; SCQ, Social Communication Questionnaire; WJ-3, Woodcock-Johnson III Tests of Cognitive Abilities, Auditory Attention subscale; NVIQ, non-verbal intelligence quotient; VIQ, verbal intelligence quotient.

**Full mutation, non-mosaic males with FXS only**

| | | Spearman's ρ | | | | Age-Corrected | |
|---|---|---|---|---|---|---|---|
| Measure | Frequency | Region | n | rho | p | rho | p |
| ABC-Abnormal Speech | theta | LPF | 24 | -.48 | 0.02 | -.50 | 0.01 |
| | | RPF | 24 | -.48 | 0.02 | -.49 | 0.02 |
| | alpha1 | LPF | 24 | -.50 | 0.01 | -.49 | 0.02 |
| | | RPF | 24 | -.44 | 0.03 | -.42 | 0.05 |
| | gamma1 | LPF | 24 | .36 | 0.08 | .42 | 0.05 |
| | | RF | 24 | .46 | 0.03 | .50 | 0.02 |
| | | RL | 24 | .44 | 0.03 | .46 | 0.03 |
| | | RP | 24 | .46 | 0.02 | .49 | 0.02 |
| | | RPF | 24 | .46 | 0.02 | .50 | 0.02 |
| | | RT | 24 | .39 | 0.06 | .47 | 0.02 |
| | gamma2 | LL | 24 | .43 | 0.04 | .48 | 0.02 |
| | | LPF | 24 | .38 | 0.07 | .45 | 0.03 |
| | | RC | 24 | .39 | 0.06 | .45 | 0.03 |
| | | RL | 24 | .44 | 0.03 | .47 | 0.02 |
| | | RP | 24 | .57 | <0.01 | .60 | <0.01 |
| | | RPF | 24 | .54 | <0.01 | .58 | <0.01 |
| | | RT | 24 | .54 | <0.01 | .59 | <0.01 |
| ABC-Hyperactivity | gamma1 | LF | 24 | .08 | 0.72 | .43 | 0.04 |
| | | RP | 24 | .26 | 0.23 | .42 | 0.04 |
| ABC-Irritability | | | 24 | .34 | 0.1 | .42 | 0.04 |
| ABC-Lethargy | theta | LPF | 24 | -.46 | 0.02 | -.48 | 0.02 |
| | | RPF | 24 | -.49 | 0.02 | -.49 | 0.02 |
| | alpha1 | LPF | 24 | -.46 | 0.02 | -.45 | 0.03 |
| | | RPF | 24 | -.53 | <0.01 | -.51 | 0.01 |
| | alpha2 | LPF | 24 | -.46 | 0.02 | -.44 | 0.04 |
| | | RPF | 24 | -.51 | 0.01 | -.49 | 0.02 |
| | gamma1 | LF | 24 | .33 | 0.11 | .43 | 0.04 |
| ABC-Stereotypy | theta | LC | 24 | -.33 | 0.12 | -.41 | 0.05 |
| | | LF | 24 | -.40 | 0.05 | -.49 | 0.02 |
| | | LPF | 24 | -.53 | <0.01 | -.60 | <0.01 |
| | | RC | 24 | -.33 | 0.11 | -.45 | 0.03 |
| | | RP | 24 | -.30 | 0.15 | -.43 | 0.04 |
| | | RPF | 24 | -.55 | <0.01 | -.59 | <0.01 |
| | | RT | 24 | -.40 | 0.06 | -.43 | 0.04 |
| | alpha1 | LPF | 24 | -.64 | <0.01 | -.66 | <0.01 |
| | | RO | 24 | -.51 | 0.01 | -.43 | 0.04 |
| | | RPF | 24 | -.62 | <0.01 | -.62 | <0.01 |
| | alpha2 | LPF | 24 | -.56 | <0.01 | -.55 | <0.01 |
| | | RPF | 24 | -.51 | 0.01 | -.48 | 0.02 |
| ADAMS-Anxiety | alpha1 | | 27 | -.48 | 0.01 | -.46 | 0.02 |
| | | RT | 27 | -.45 | 0.02 | -.39 | 0.05 |

| | | Spearman's ρ | | | | Age-Corrected | |
|---|---|---|---|---|---|---|---|
| Measure | Frequency | Region | n | rho | p | rho | p |
| | alpha2 | LO | 27 | -.49 | <0.01 | -.43 | 0.03 |
| | | LPF | 27 | -.49 | <0.01 | -.47 | 0.02 |
| | | LT | 27 | -.58 | <0.01 | -.54 | <0.01 |
| | | RO | 27 | -.60 | <0.01 | -.56 | <0.01 |
| | | RPF | 27 | -.48 | 0.01 | -.46 | 0.02 |
| | | RT | 27 | -.48 | 0.01 | -.42 | 0.03 |
| ADAMS-OCD | alpha1 | LO | 27 | -.53 | <0.01 | -.45 | 0.02 |
| | | LT | 27 | -.56 | <0.01 | -.48 | 0.01 |
| | | RO | 27 | -.47 | 0.01 | -.40 | 0.04 |
| NVIQ | alpha2 | LT | 22 | -.50 | 0.02 | -.46 | 0.04 |
| | gamma1 | LP | 22 | -.45 | 0.04 | -.44 | 0.04 |
| | | LT | 22 | -.51 | 0.01 | -.48 | 0.03 |
| | | RO | 22 | -.62 | <0.01 | -.60 | <0.01 |
| | | RT | 22 | -.52 | 0.01 | -.49 | 0.02 |
| | gamma2 | LP | 22 | -.47 | 0.03 | -.46 | 0.04 |
| | | LT | 22 | -.53 | 0.01 | -.51 | 0.02 |
| | | RO | 22 | -.59 | <0.01 | -.57 | <0.01 |
| | | RT | 22 | -.55 | <0.01 | -.54 | 0.01 |
| SCQ | alpha1 | RPF | 24 | -.44 | 0.03 | -.43 | 0.04 |
| | alpha2 | | 24 | -.49 | 0.01 | -.46 | 0.03 |
| VIQ | | LT | 22 | -.30 | 0.17 | -.57 | <0.01 |
| | | RT | 22 | -.25 | 0.26 | -.46 | 0.04 |
| WJ-III | theta | LO | 23 | .32 | 0.14 | .45 | 0.04 |
| | | LP | 23 | .42 | 0.05 | .46 | 0.03 |
| | | RO | 23 | .31 | 0.15 | .45 | 0.04 |
| | | RP | 23 | .38 | 0.07 | .52 | 0.01 |
| | alpha1 | LO | 23 | .60 | <0.01 | .52 | 0.01 |
| | gamma1 | | 23 | -.17 | 0.44 | -.43 | 0.05 |
| | | RP | 23 | -.41 | 0.05 | -.49 | 0.02 |
| | gamma2 | LO | 23 | -.22 | 0.31 | -.48 | 0.02 |
| | | RO | 23 | -.29 | 0.18 | -.43 | 0.04 |
| | | RP | 23 | -.41 | 0.05 | -.49 | 0.02 |

**Supplementary Table 8. Age-corrected Clinical Correlations of Spectral Power by Resting State Network (RSN)**

Abbreviations: ABC, Aberrant Behavior Checklist; ADAMS, Anxiety, Depression and Mood Scale; SCQ, Social Communication Questionnaire; WJ-3, Woodcock-Johnson III Tests of Cognitive Abilities, Auditory Attention subscale; Map, RSN or region label; DMN, default mode network; DAN, dorsal attention network; SAN, salient affective network; VIS, visual attention network; AUD, auditory network.

**All FXS Participants**

| | | Spearman's ρ | | | | Age-Corrected | |
|---|---|---|---|---|---|---|---|
| Measure | Frequency | RSN | n | rho | adj.p | rho | adj.p |
| ABC-Abnormal Speech | gamma2 | SAN | 61 | .38 | 0.03 | .36 | 0.05 |
| ABC-Hyperactivity | alpha1 | AUD | 61 | -.46 | 0.01 | -.44 | 0.02 |
| | | DAN | 61 | -.42 | 0.02 | -.40 | 0.03 |
| ABC-Stereotypy | theta | AUD | 61 | -.38 | 0.03 | -.38 | 0.04 |
| | alpha1 | | 61 | -.36 | 0.03 | -.40 | 0.03 |
| | | DAN | 61 | -.36 | 0.03 | -.39 | 0.03 |
| | | VIS | 61 | -.36 | 0.03 | -.44 | 0.02 |
| | alpha2 | DAN | 61 | -.34 | 0.04 | -.37 | 0.04 |
| | | DMN | 61 | -.34 | 0.04 | -.39 | 0.03 |
| | | VIS | 61 | -.30 | 0.08 | -.38 | 0.04 |
| ADAMS-OCD | alpha1 | AUD | 64 | -.45 | 0.01 | -.46 | 0.02 |
| | | DAN | 64 | -.38 | 0.03 | -.38 | 0.03 |
| | | DMN | 64 | -.39 | 0.02 | -.41 | 0.03 |
| | | VIS | 64 | -.39 | 0.02 | -.42 | 0.02 |
| | alpha2 | AUD | 64 | -.41 | 0.02 | -.42 | 0.02 |
| | | DAN | 64 | -.38 | 0.03 | -.38 | 0.03 |
| | | DMN | 64 | -.50 | <0.01 | -.52 | <0.01 |
| | | SAN | 64 | -.41 | 0.02 | -.42 | 0.02 |
| | | VIS | 64 | -.33 | 0.04 | -.36 | 0.05 |
| SCQ | alpha1 | AUD | 62 | -.32 | 0.05 | -.36 | 0.05 |
| | | DAN | 62 | -.37 | 0.03 | -.40 | 0.03 |
| | | DMN | 62 | -.34 | 0.04 | -.40 | 0.03 |
| | | SAN | 62 | -.33 | 0.05 | -.36 | 0.05 |
| | alpha2 | DAN | 62 | -.36 | 0.03 | -.39 | 0.03 |
| | | DMN | 62 | -.39 | 0.03 | -.44 | 0.02 |
| | | SAN | 62 | -.39 | 0.02 | -.41 | 0.03 |
| WJ-III | alpha1 | | 58 | .37 | 0.03 | .39 | 0.03 |

**Supplementary Table 9. Age-corrected Clinical Correlations of Spectral Power by Resting State Network**

Abbreviations: ABC, Aberrant Behavior Checklist; ADAMS, Anxiety, Depression and Mood Scale; WJ-3, Woodcock-Johnson III Tests of Cognitive Abilities, Auditory Attention subscale; NVIQ, non-verbal intelligence quotient; Map, RSN or region label; DMN, default mode network; DAN, dorsal attention network; SAN, salient affective network; VIS, visual attention network; AUD, auditory network.

**Full mutation, non-mosaic males with FXS only**

| | | Spearman's ρ | | | | Age-Corrected | |
|---|---|---|---|---|---|---|---|
| Measure | Frequency | Rsn | n | rho | p | rho | p |
| ABC-Abnormal Speech | alpha2 | SAN | 24 | .37 | 0.08 | .51 | 0.01 |
| | gamma1 | AUD | 24 | .34 | 0.1 | .43 | 0.04 |
| | | DAN | 24 | .42 | 0.04 | .51 | 0.01 |
| | | DMN | 24 | .39 | 0.06 | .43 | 0.04 |
| | | SAN | 24 | .40 | 0.05 | .45 | 0.03 |
| | gamma2 | AUD | 24 | .44 | 0.03 | .49 | 0.02 |
| | | DAN | 24 | .46 | 0.02 | .53 | <0.01 |
| | | DMN | 24 | .46 | 0.02 | .51 | 0.01 |
| ABC-Lethargy | theta | AUD | 24 | -.41 | 0.05 | -.45 | 0.03 |
| ABC-Stereotypy | | | 24 | -.37 | 0.08 | -.47 | 0.02 |
| | | DAN | 24 | -.38 | 0.07 | -.43 | 0.04 |
| | | DMN | 24 | -.46 | 0.02 | -.46 | 0.03 |
| | | SAN | 24 | -.39 | 0.06 | -.47 | 0.03 |
| | alpha1 | DAN | 24 | -.47 | 0.02 | -.44 | 0.04 |
| ADAMS-Anxiety | alpha2 | VIS | 27 | -.55 | <0.01 | -.51 | <0.01 |
| ADAMS-OCD | alpha1 | AUD | 27 | -.49 | <0.01 | -.44 | 0.03 |
| | | VIS | 27 | -.51 | <0.01 | -.41 | 0.04 |
| | alpha2 | DMN | 27 | -.50 | <0.01 | -.40 | 0.04 |
| NVIQ | gamma1 | AUD | 22 | -.49 | 0.02 | -.45 | 0.04 |
| | | DAN | 22 | -.48 | 0.02 | -.44 | 0.04 |
| | | DMN | 22 | -.47 | 0.03 | -.46 | 0.04 |
| | | VIS | 22 | -.63 | <0.01 | -.60 | <0.01 |
| | gamma2 | AUD | 22 | -.54 | <0.01 | -.52 | 0.02 |
| | | DMN | 22 | -.45 | 0.03 | -.44 | 0.05 |
| | | VIS | 22 | -.53 | 0.01 | -.50 | 0.02 |
| WJ-III | gamma1 | | 23 | -.23 | 0.3 | -.42 | 0.05 |
| | gamma2 | | 23 | -.25 | 0.25 | -.44 | 0.04 |

**Supplementary Table 10. Age-corrected Clinical Correlations of Peak Alpha Frequency (PAF) by Resting State Network (RSN) in All FXS Participants (trending significance following FDR correction).**

Abbreviations: VIQ, verbal intelligence quotient; SAN, salient affective network.

| Measure | RSN | Type | r | p | adj. p | n |
|---|---|---|---|---|---|---|
| VIQ | SAN | PAF | .39 | 1.5e-03 | 0.081 | 64 |

**Supplementary Table 11. Age-corrected Clinical Correlations of Peak Alpha Frequency (PAF) by Cortical Region in full mutation, non-mosaic males with FXS.**

Abbreviations: ABC, Aberrant Behavior Checklist; ADAMS, Anxiety, Depression and Mood Scale. Cortical regions include occipital (O), Limbic (L), parietal (P), temporal (T), central (C), frontal (F), and prefrontal (PF) each with a preceding right (R) or left (L) designation.

| | Spearman's ρ | | | | Age-Corrected | |
|---|---|---|---|---|---|---|
| Measure | Region | n | rho | p | rho | p |
| ABC-Hyperactivity | LC | 24 | .14 | 0.53 | .42 | 0.05 |
| | LF | 24 | -.50 | 0.01 | -.48 | 0.02 |
| | LO | 24 | -.54 | <0.01 | -.42 | 0.05 |
| ABC-Stereotypy | | 24 | -.54 | <0.01 | -.45 | 0.03 |
| | RPF | 24 | .54 | <0.01 | .54 | <0.01 |
| ADAMS-Anxiety | LO | 27 | -.57 | <0.01 | -.53 | <0.01 |
| | LPF | 27 | -.48 | 0.01 | -.43 | 0.03 |
| | LT | 27 | -.53 | <0.01 | -.53 | <0.01 |
| | RP | 27 | -.42 | 0.03 | -.41 | 0.04 |
| | RT | 27 | -.42 | 0.03 | -.46 | 0.02 |

**Supplementary Table 12. Age-corrected Clinical Correlations of Peak Alpha Frequency (PAF) by Resting State Network (RSN) in full mutation, non-mosaic males with FXS.**

Abbreviations: ADAMS, Anxiety, Depression and Mood Scale; DMN, Default Mode Network.

| | | Spearman's ρ | | | Age-Corrected | |
|---|---|---|---|---|---|---|
| Measure | RSN | n | rho | p | rho | p |
| ADAMS-Anxiety | DMN | 27 | -.44 | 0.02 | -.46 | 0.02 |

**Supplementary Table 13. Age-corrected Clinical Correlations of Power-Power Cross-frequency Coupling (CFC) by Cortical Region in full mutation, non-mosaic males with FXS.** No significant correlations were present across all FXS participants following FDR correction.

Abbreviations: ABC, Aberrant Behavior Checklist; ADAMS, Anxiety, Depression and Mood Scale; NVIQ, non-verbal intelligence quotient; VIQ, verbal intelligence quotient; WJ-III, Woodcock Johnson III Test of Auditory Attention. Cortical regions include occipital (O), Limbic (L), parietal (P), temporal (T), central (C), frontal (F), and prefrontal (PF) each with a preceding right (R) or left (L) designation.

| | | Spearman's ρ | | | | Age-Corrected | |
|---|---|---|---|---|---|---|---|
| Measure | Frequency | Region | n | rho | p | rho | p |
| ADAMS-Anxiety | alpha1 | RO | 27 | .43 | 0.02 | .44 | 0.03 |
| ADAMS-OCD | theta | | 27 | .40 | 0.04 | .39 | 0.05 |
| | alpha1 | | 27 | .40 | 0.04 | .42 | 0.03 |
| NVIQ | theta | LF | 22 | .49 | 0.02 | .45 | 0.04 |
| | | LT | 22 | .55 | <0.01 | .51 | 0.02 |
| | alpha1 | RO | 22 | .58 | <0.01 | .58 | <0.01 |
| | | RT | 22 | .60 | <0.01 | .58 | <0.01 |
| | alpha2 | RO | 22 | .44 | 0.04 | .44 | 0.04 |
| | | RP | 22 | .42 | 0.05 | .44 | 0.04 |
| | | RT | 22 | .56 | <0.01 | .55 | <0.01 |
| VIQ | alpha1 | LT | 22 | -.53 | 0.01 | -.50 | 0.02 |
| | alpha2 | | 22 | -.56 | <0.01 | -.55 | <0.01 |
| WJ-III | | RC | 23 | .42 | 0.05 | .47 | 0.03 |

**Supplementary Table 14. Age-corrected Clinical Correlations of Power-Power Cross-frequency Coupling (CFC) by Resting State Network (RSN) in full mutation, non-mosaic males with FXS.** No significant correlations were present across all FXS participants following FDR correction.

Abbreviations: ADAMS, Anxiety, Depression and Mood Scale; OCD, obsessive compulsive disorder; NVIQ, non-verbal intelligence quotient. Resting state networks include default mode network (DMN), dorsal attention network (DAN), salient affective network (SAN), auditory network (AUD), and visual network (VIS).

| | | Spearman's ρ | | | | Age-Corrected | |
|---|---|---|---|---|---|---|---|
| Measure | Frequency | Rsn | n | rho | p | rho | p |
| ADAMS-Anxiety | alpha1 | VIS | 27 | .42 | 0.03 | .41 | 0.04 |
| ADAMS-OCD | alpha2 | | 27 | .38 | 0.05 | .40 | 0.04 |
| NVIQ | theta | DAN | 22 | .47 | 0.03 | .44 | 0.05 |
| | | SAN | 22 | .52 | 0.01 | .49 | 0.03 |
| | alpha1 | AUD | 22 | .48 | 0.02 | .46 | 0.04 |
| | | DAN | 22 | .54 | <0.01 | .53 | 0.01 |
| | | VIS | 22 | .45 | 0.03 | .44 | 0.04 |
| | alpha2 | DAN | 22 | .48 | 0.02 | .46 | 0.04 |

**Supplementary Table 15. Assignment of cortical nodes to region and resting state network (RSN) groups as attributed by the Desikan-Killiany (DK) atlas.** The MNI coordinates and number of vertices included in each node parcel are also displayed. Region and RSN represents an anatomical and functional grouping strategy for cortical nodes respectively.

Abbreviations: RSN, resting state networks; DMN, default mode network; DAN, dorsal attention network; SAN, salience affective network; VIS, visual network; other, unassigned nodes. L, Left; R, right; F, frontal; L, limbic; O, occipital; P, parietal; PF, prefrontal; T, temporal.

| | | | | MNI Centroid | | | |
|---|---|---|---|---|---|---|---|
| RSN | Node | Abbreviation | Cortex | x | y | z | Vert. |
| DMN | Caudal Anterior Cingulate R | cACC R | RL | 4 | 22 | 27 | 68 |
| | Isthmus Cingulate L | iCC L | LL | -7 | -45 | 18 | 81 |
| | Isthmus Cingulate R | iCC R | RL | 6 | -42 | 19 | 91 |
| | Lateral Orbitofrontal L | LOF L | LPF | -25 | 30 | -19 | 221 |
| | Lateral Orbitofrontal R | LOF R | RPF | 23 | 32 | -19 | 206 |
| | Medial Orbitofrontal L | MOF L | LPF | -7 | 33 | -17 | 155 |
| | Medial Orbitofrontal R | MOF R | RPF | 4 | 37 | -15 | 170 |
| | Parahippocampal L | paraH L | LT | -26 | -29 | -21 | 68 |
| | Parahippocampal R | paraH R | RT | 24 | -30 | -19 | 66 |
| | Posterior Cingulate L | PCC L | LL | -5 | -15 | 38 | 85 |
| | Posterior Cingulate R | PCC R | RL | 5 | -17 | 39 | 93 |
| | Precuneus L | PCUN L | LP | -9 | -59 | 38 | 314 |
| | Precuneus R | PCUN R | RP | 10 | -58 | 38 | 325 |
| | Rostral Anterior Cingulate L | rACC L | LL | -5 | 39 | 1 | 78 |
| | Rostral Anterior Cingulate R | rACC R | RL | 4 | 38 | 3 | 56 |
| SAN | Caudal Middle Frontal L | cMFG L | LF | -37 | 11 | 47 | 224 |
| | Caudal Middle Frontal R | cMFG R | RF | 37 | 13 | 48 | 186 |
| | Insula L | INS L | LT | -38 | -2 | 2 | 174 |
| | Insula R | INS R | RT | 36 | 2 | -2 | 196 |
| | Rostral Middle Frontal L | rMFG L | LF | -34 | 47 | 17 | 543 |
| | Rostral Middle Frontal R | rMFG R | RF | 34 | 48 | 17 | 551 |
| | Supramarginal L | SMAR L | LP | -57 | -38 | 34 | 305 |
| | Supramarginal R | SMAR R | RP | 54 | -31 | 36 | 302 |
| DAN | Caudal Anterior Cingulate L | cACC L | LL | -5 | 21 | 26 | 48 |
| | Inferior Temporal L | ITG L | LT | -53 | -36 | -22 | 307 |
| | Inferior Temporal R | ITG R | RT | 51 | -32 | -25 | 316 |
| | Middle Temporal L | MTG L | LT | -58 | -23 | -15 | 277 |
| | Middle Temporal R | MTG R | RT | 58 | -22 | -15 | 324 |
| | Pars Opercularis L | pOPER L | LF | -49 | 17 | 14 | 139 |
| | Pars Opercularis R | pOPER R | RF | 49 | 17 | 14 | 118 |
| | Pars Orbitalis L | pORB L | LPF | -44 | 39 | -14 | 72 |
| | Pars Orbitalis R | pORB R | RPF | 43 | 42 | -15 | 68 |
| | Pars Triangularis L | pTRI L | LF | -47 | 32 | 1 | 101 |
| | Pars Triangularis R | pTRI R | RF | 48 | 34 | 2 | 148 |
| AUD | Superior Temporal L | STG L | LT | -55 | -12 | -4 | 290 |
| | Superior Temporal R | STG R | RT | 54 | -6 | -7 | 257 |
| VIS | Cuneus L | CUN L | LO | -6 | -80 | 19 | 93 |
| | Cuneus R | CUN R | RO | 8 | -78 | 20 | 99 |
| | Fusiform L | FUS L | LT | -36 | -43 | -22 | 268 |
| | Fusiform R | FUS R | RT | 35 | -41 | -23 | 255 |

| | | | | MNI Centroid | | | |
|---|---|---|---|---|---|---|---|
| RSN | Node | Abbreviation | Cortex | x | y | z | Vert. |
| | Lateral Occipital L | LOG L | LO | -31 | -89 | 0 | 371 |
| | Lateral Occipital R | LOG R | RO | 35 | -85 | 2 | 367 |
| | Lingual L | LING L | LO | -14 | -71 | -5 | 246 |
| | Lingual R | LING R | RO | 13 | -67 | -4 | 227 |
| other | Banks of Sup. Temp. Sulcus L | BSTS L | LT | -53 | -45 | 8 | 76 |
| | Banks of Sup. Temp. Sulcus R | BSTS R | RT | 54 | -41 | 10 | 70 |
| | Entorhinal L | ENT L | LT | -26 | -5 | -33 | 30 |
| | Entorhinal R | ENT R | RT | 23 | -6 | -35 | 32 |
| | Frontal Pole L | FP L | LPF | -7 | 68 | -11 | 22 |
| | Frontal Pole R | FP R | RPF | 7 | 68 | -15 | 30 |
| | Inferior Parietal L | IPL L | LP | -42 | -71 | 32 | 351 |
| | Inferior Parietal R | IPL R | RP | 46 | -63 | 32 | 421 |
| | Paracentral L | paraC L | LC | -7 | -30 | 57 | 110 |
| | Paracentral R | paraC R | RC | 7 | -27 | 57 | 128 |
| | Pericalcarine L | periCAL L | LO | -11 | -82 | 6 | 109 |
| | Pericalcarine R | periCAL R | RO | 12 | -80 | 7 | 110 |
| | Postcentral L | postC L | LC | -46 | -22 | 45 | 333 |
| | Postcentral R | postC R | RC | 44 | -20 | 46 | 307 |
| | Precentral L | preC L | LC | -41 | -9 | 46 | 339 |
| | Precentral R | preC R | RC | 40 | -7 | 46 | 353 |
| | Superior Frontal L | sFG L | LF | -12 | 30 | 41 | 671 |
| | Superior Frontal R | sFG R | RF | 12 | 32 | 41 | 603 |
| | Superior Parietal L | SPL L | LP | -23 | -65 | 50 | 484 |
| | Superior Parietal R | SPL R | RP | 24 | -65 | 51 | 464 |
| | Temporal Pole L | TP L | LT | -28 | 14 | -38 | 38 |
| | Temporal Pole R | TP R | RT | 27 | 16 | -36 | 38 |
| | Transverse Temporal L | TT L | LT | -46 | -23 | 10 | 34 |
| | Transverse Temporal R | TT R | RT | 46 | -17 | 9 | 23 |

## Supplementary References

1. B, B., et al., *Spatial and Temporal Resolutions of EEG: Is It Really Black and White? A Scalp Current Density View.* International journal of psychophysiology : official journal of the International Organization of Psychophysiology, 2015. **97**(3).
