# Supplementary material for: Neocortical localization and thalamocortical modulation of neuronal hyperexcitability contribute to Fragile X Syndrome
Source: Commun Biol. 2022 May 11;5:442. doi: 10.1038/s42003-022-03395-9 (PMC9095835; doi:10.1038/s42003-022-03395-9)
Supplement: Supplementary file 3 — Reporting Summary [file 42003_2022_3395_MOESM3_ESM.pdf]

nature research

Corresponding author(s): Ernest Pedapati, MD

Last updated by author(s): Mar 28, 2022

Reporting Summary

Nature Research wishes to improve the reproducibility of the work that we publish. This form provides structure for consistency and transparency in reporting. For further information on Nature Research policies, see our Editorial Policies and the Editorial Policy Checklist.

Statistics

For all statistical analyses, confirm that the following items are present in the figure legend, table legend, main text, or Methods section.

n/a	Confirmed	
☐	☒	The exact sample size (*n*) for each experimental group/condition, given as a discrete number and unit of measurement
☐	☒	A statement on whether measurements were taken from distinct samples or whether the same sample was measured repeatedly
☐	☒	The statistical test(s) used AND whether they are one- or two-sided<br>*Only common tests should be described solely by name; describe more complex techniques in the Methods section.*
☐	☒	A description of all covariates tested
☐	☒	A description of any assumptions or corrections, such as tests of normality and adjustment for multiple comparisons
☐	☒	A full description of the statistical parameters including central tendency (e.g. means) or other basic estimates (e.g. regression coefficient) AND variation (e.g. standard deviation) or associated estimates of uncertainty (e.g. confidence intervals)
☐	☒	For null hypothesis testing, the test statistic (e.g. *F*, *t*, *r*) with confidence intervals, effect sizes, degrees of freedom and *P* value noted<br>*Give P values as exact values whenever suitable.*
☒	☐	For Bayesian analysis, information on the choice of priors and Markov chain Monte Carlo settings
☒	☐	For hierarchical and complex designs, identification of the appropriate level for tests and full reporting of outcomes
☐	☒	Estimates of effect sizes (e.g. Cohen's *d*, Pearson's *r*), indicating how they were calculated

Our web collection on statistics for biologists contains articles on many of the points above.

Software and code

Policy information about availability of computer code

Data collection	Acquisition of EEG data was performed in EGI/Phillips Netstation 5.0 (EGI, Eugene, OR, USA).
Data analysis	Statistical analysis was performed with MATLAB 2018b (Mathworks, Natick, MA, USA), SAS 9.4 (SAS Institute Inc., Cary, NC, USA), and R (4.1, Vienna, Austria). Datasets and analysis code repository accession numbers are included in the manuscript. Scripts used for EEG analysis are available at http://github.com/cincibrainlab/vhtp. Time-stamped analysis code is available on Figshare at https://doi.org/10.6084/m9.figshare.19424015.v12.

For manuscripts utilizing custom algorithms or software that are central to the research but not yet described in published literature, software must be made available to editors and reviewers. We strongly encourage code deposition in a community repository (e.g. GitHub). See the Nature Research guidelines for submitting code & software for further information.

Data

Policy information about availability of data

All manuscripts must include a data availability statement. This statement should provide the following information, where applicable:

- Accession codes, unique identifiers, or web links for publicly available datasets
- A list of figures that have associated raw data
- A description of any restrictions on data availability

EEG dataset analyzed in this study are openly available in Zenodo at https://doi.org/10.5281/zenodo.6385768.
Source data for figures are available in Figshare at https://doi.org/10.6084/m9.figshare.19424015.v12.

Field-specific reporting

Please select the one below that is the best fit for your research. If you are not sure, read the appropriate sections before making your selection.

☒ Life sciences ☐ Behavioural & social sciences ☐ Ecological, evolutionary & environmental sciences

For a reference copy of the document with all sections, see nature.com/documents/nr-reporting-summary-flat.pdf

Life sciences study design

All studies must disclose on these points even when the disclosure is negative.

Sample size	Differences in gamma1 power in FXS compared to controls in previous studies have effect sizes from .63 to 1.75, similar to effect sizes in prior studies of N1 amplitudes in FXS6, 13, 23, 75. Based on these effect sizes, comparing 70 FXS patients (50% males) and 70 TD controls provides power to detect the primary EEG outcome with approximately power > .90 (using an omnibus F-test with an alpha of .05).
Data exclusions	The dataset included a total of 145 participants drawn from a large federally funded human neurophysiology study in FXS (National Institutes of Mental Health U54 HD082008). Following blinded preprocessing, three recordings were discarded from further analysis due to excessive line-noise artifact (1 FXS, 2 controls) and one due to insufficient data due to intolerance of the EEG procedure (1 FXS).
Replication	As noted in the references (Wang 2017) we have extended our key findings in the present sample, which includes a new cohort of subjects, new location of recruitment, and saline-based EEG nets versus gel-based nets.
Randomization	Recruitment of human subjects was based on diagnostic categorization. This was a case-control study, no randomization of subjects for an intervention was performed.
Blinding	All data was blinded and coded in regard to diagnostic group participant, or collection date for preprocessing and analysis. Groups were unblinded following analysis.

Reporting for specific materials, systems and methods

We require information from authors about some types of materials, experimental systems and methods used in many studies. Here, indicate whether each material, system or method listed is relevant to your study. If you are not sure if a list item applies to your research, read the appropriate section before selecting a response.

Materials & experimental systems

n/a	Involved in the study	
☒	☐	Antibodies
☒	☐	Eukaryotic cell lines
☒	☐	Palaeontology and archaeology
☒	☐	Animals and other organisms
☐	☒	Human research participants
☒	☐	Clinical data
☒	☐	Dual use research of concern

Methods

n/a	Involved in the study	
☒	☐	ChIP-seq
☒	☐	Flow cytometry
☒	☐	MRI-based neuroimaging

Human research participants

Policy information about studies involving human research participants

Population characteristics	The dataset included a total of 145 participants drawn from a large federally funded human neurophysiology study in FXS (National Institutes of Mental Health U54 HD082008). Exclusion criteria for FXS participants (confirmed by Southern Blot and polymerase chain reaction) included present history of unstable seizures (any treated seizure within one year) and scheduled use of benzodiazepines. Controls did not have treatment for neuropsychiatric illness as reported via clinical interview.
Recruitment	All participants were recruited through medical clinics, electronic email solicitation, or community based fliers or social media advertising consistent with the approved IRB approval.
Ethics oversight	All participants provided written informed consent (or assent as appropriate) prior to participation as approved by the institutional review board of Cincinnati Children’s Hospital Medical Center. A Data Safety Monitoring Board met regularly for oversight of this study.

Note that full information on the approval of the study protocol must also be provided in the manuscript.
